# Integrating Early Transcriptomic Responses to Rhizotoxins in Rice (*Oryza sativa.* L.) Reveals Key Regulators and a Potential Early Biomarker of Cadmium Toxicity

**DOI:** 10.3389/fpls.2017.01432

**Published:** 2017-08-18

**Authors:** Li-Yao Huang, Chung-Wen Lin, Ruey-Hua Lee, Chih-Yun Chiang, Yung-Chuan Wang, Ching-Han Chang, Hao-Jen Huang

**Affiliations:** ^1^Department of Life Sciences, National Cheng Kung University Tainan, Taiwan; ^2^Institute of Tropical Plant Sciences, National Cheng Kung University Tainan, Taiwan

**Keywords:** cadmium biomarker, early transcriptomic response, gene architecture, *Oryza sativa* L., regulatory network, rhizotoxins, heavy metal

## Abstract

As sessile organisms, plants were constantly challenged with biotic and abiotic stresses. Transcriptional activation of stress-responsive genes is a crucial part of the plant adaptation to environmental changes. Here, early response of rice root to eight rhizotoxic stressors: arsenate, copper, cadmium, mercury, chromate, vanadate, ferulic acid and juglone, was analyzed using published microarray data. There were 539 general stress response (GSR) genes up-regulated under all eight treatments, including genes related to carbohydrate metabolism, phytohormone balance, and cell wall structure. Genes related to transcriptional coactivation showed higher *K*_a_*/K*_s_ ratio compared to the other GSR genes. Network analysis discovered complicated interaction within GSR genes and the most connected signaling hubs were *WRKY53, WRKY71*, and *MAPK5*. Promoter analysis discovers enriched SCGCGCS *cis*-element in GSR genes. Moreover, GSR genes tend to be intronless and genes with shorter total intron length were induced in a higher level. Among genes uniquely up-regulated by a single stress, a phosphoenolpyruvate carboxylase kinase (PPCK) was identified as a candidate biomarker for detecting cadmium contamination. Our findings provide insights into the transcriptome dynamics of molecular response of rice to different rhizotoxic stress and also demonstrate potential use of comparative transcriptome analysis in identifying a novel potential early biomarker.

## Introduction

Soil contamination by rhizotoxins is a threat to food security (Lobell et al., 2009). Rhizotoxins in agricultural soil, such as heavy metal, metalloid and organic pollutants from sewage water and industrial waste contamination, present problem for normal root function leading to reduced crop yields. The most common heavy metal pollutants found in soil for plants are Cd, Pb, Cr, Hg, As, Cu, Ni, and Zn (Schützendübel and Polle, 2002; Li et al., 2014; Ullah et al., 2015). The inhibitory effects of these heavy metals/metalloids on plant growth have been extensively studied (Hossain and Komatsu, 2012). In addition, allelopathy of plant-produced phytochemicals also can have negative effects on growth of nearby plants. It has been shown that uptake of juglone and ferulic acid can inhibit root growth (Chi et al., 2011, 2013). Accumulation of rhizotoxins in crops is a threat to food security to world populations (Tripathi et al., 2007; Meharg et al., 2013). Given the variety of rhizotoxins, it is important to understand the general stress response (GSR) to rhizotoxins in plants, especially in crops.

Plants counteract environmental stresses beginning with stress perception and signal transduction, followed by activation of a core set of functional genes (Bjornson et al., 2016). A burst of reactive oxygen species (ROS) and elevated cytosolic calcium ion concentrations are triggered immediately after stress exposure, acting as an alarm signal which is transduced by kinases such as calcium-dependent protein kinases (CDPKs) and mitogen-activated protein kinases (MAPKs) cascades (Bjornson et al., 2016). The regulation of downstream defense genes largely depends on the type of transcription factor binding site (TFBS) in the promoter region. Certain TFBSs are critical for genes to be induced under stress, and are significantly more abundant than expected by chance among a set of stress-responsive genes (Eulgem and Somssich, 2007).

Besides TFBSs, gene architecture, especially the size of genes, is another important parameter in relation to gene regulation. In plants, highly expressed genes were reported to be the least compact than the genes expressed at a low level (Ren et al., 2006). However, contradiction emerged as different results were obtained when assessing genomic data of different plants (Woody and Shoemaker, 2011). Moreover, discovery of the relationship between gene compactness and temporal expression pattern has made regulation through gene architecture more dynamic and complicated (Jeffares et al., 2008). The environmental stress may also have a significant impact on the evolutionary and ecological processes that affect and shape the genetic structure and evolution of populations (Chapin et al., 1993; Pierce et al., 2005). These investigations have led to increased interest in studying the role of environmental stress in relation to natural selection for stress resistance and adaptation.

Comparative transcriptomics combining systems biology enables us to reveal similarity and difference at molecular level when compare dynamics of molecular responses between different abiotic stresses (Cramer et al., 2011). Many studies have investigated molecular mechanisms of plant responses to multiple abiotic stresses in Arabidopsis (Swindell, 2006; Kilian et al., 2007; Urano et al., 2010). For example, Zhao et al. (2009) have identified glutathione-*S*-transferases, peroxidases, Ca-binding proteins and a trehalose-synthesizing enzyme being the core genes in response to aluminum (Al) ions, cadmium (Cd) ions, copper (Cu) ions and sodium chloride (NaCl) stresses. They also identified known resistant genes specific to Al (*AtALMT1*, Al-activated malate transporter) and NaCl (*AtDREB*, dehydration responsive element binding protein). Results from studies in the filed shed light on crop improvement strategy against single and multiple stressors (Cabello et al., 2014; Mickelbart et al., 2015). Researching on GSR using a transcriptomic approach may lead to discovery of uniquely up-regulated genes, which are potent candidate biomarkers for detecting particular environmental stresses. The idea of using biomarkers from terrestrial plants to detect soil contamination was proposed two decades ago. Gene expression, metabolite level, and enzyme activity, which show differential regulation after stress exposure, have been proposed as candidate biomarkers (Feder and Hofmann, 1999; Ernst et al., 2000; da Rosa Corrêa et al., 2006). However, despite plenty of candidates being proposed over the past decades, their practical uses have been challenged by field conditions where multiple stresses frequently occur in combinations (Rizhsky et al., 2002). To our knowledge, to date there is no applicable biomarker that holds its specificity to a particular heavy metal stress under a multi-stress environment.

Our previous works have revealed early molecular responses (1 and 3 h) of rice to individual rhizotoxins (Chi et al., 2011, 2013; Huang et al., 2012, 2014; Lin et al., 2013a,b; Chen et al., 2014). In the present study, we focused on the early GSR of rice by integrating previously published data. A large-scale transcriptome analysis was conducted to study the early response of rice root to eight rhizotoxic stressors: arsenate (As), copper (Cu), cadmium (Cd), mercury (Hg), chromate (Cr), vanadate (V), ferulic acid (FA), and juglone. We revealed rapidly induced GSR genes as well as uniquely up-regulated genes upon exposure to these rhizotoxins in rice. The higher *K*_a_*/K*_s_ ratio of genes related to transcriptional coactivation than GSR genes and the genome median demonstrates the rapid evolution of genes related to transcriptional coactivation. Interaction networks of genes were constructed to identify important hubs. The relationship between gene architecture and expression pattern was analyzed. In addition, phosphoenolpyruvate carboxylase kinase 2 (*OsPPCK2*) was found to be uniquely up-regulated by cadmium even under combined stress conditions. We consider *OsPPCK2* a candidate biomarker for detecting cadmium contamination.

## Materials and Methods

### Plant Materials

Sterilization of rice (*Oryza sativa* L. cv. TN-67) seeds was performed with 2.5% (v/v) sodium hypochlorite (Katayama, Japan) for 15 min, and then washed thoroughly in distilled water. Around 100 seeds were placed in 9 cm Petri dishes containing 20 ml distilled water at 37°C in darkness for 3 days. Seeds with uniform germination were transferred to water-soaked filter paper disk (Advantec No. 1) in 15 cm Petri dishes (30 seeds for each Petri dish) containing 20 ml distilled water, and incubated at 26°C in darkness for 3 days. Six-days-old rice seedlings in 15 cm Petri dishes were then exposed to different stresses by submerging under following solutions: 5 μM CuCl_2_ (copper), 25 μM CdCl_2_ (cadmium), 25 μM HgCl_2_ (mercury), 50 μM K_2_CrO_4_ (chromate), 25 μM Na_2_AsO_4_⋅7H_2_O (arsenic), 1 mM Na_3_VO_4_ (vanadate), 50 ppm ferulic acid (FA) or 10 μM juglone for 1 and 3 h. Control plants were treated with water in parallel for the indicated times. After treatment, root tips (2 cm) were harvested and subjected to RNA extraction. All the chemicals were purchased from Sigma (St. Louis, MO, United States).

### Microarray Data Source and Analysis

A total of eight previously published rice microarray data sets were downloaded from Gene Expression Omnibus database [(GSE IDs: GSE33375, GSE63152, GSE41719, GSE41733, GSE34899, GSE27413, and GSE34895)^1^] (Chi et al., 2011, 2013; Huang et al., 2012, 2014; Lin et al., 2013a,b; Chen et al., 2014). Each data set was composed of three biological replicates. All data sets were achieved by using identical genotype (*O. sativa* L. cv. TN-67), plant growth conditions, experiment protocols but eight different stresses. For data analysis, all data sets were normalized together with use of GeneSpringGX12 (Agilent Technologies). Genes with signal intensities < 100 were excluded. Statistical analysis was conducted with Kruskal–Wallis test. The Benjamini–Hochberg FDR method was used to obtain corrected *p*-values (false discovery rate, FDR) for multiple testing. Genes with at least twofold change (FC) in transcript abundance were considered as up- or down-regulated (FC ≥ 2 or FC ≤-2; cutoff FDR < 0.05). In addition, four gene groups were defined by distinct expression patterns: general stress response genes (GSR): FC ≥ 2 under all eight stresses; background genes: |FC|≤ 1.2 under all eight stresses; low-regulated genes: 2 > FC > 1.2 under all eight stresses; uniquely regulated genes: |FC|≥ 2 under one stress and |FC|≤ 1.4 under the others. In addition, GSR genes were manually classified into 19 different functional categories based on GOsilm categories from BiNGO^2^, functional categories from MapMan^3^ and descriptions from Rice Genome Annotation Project^4^ as well as literatures (Supplementary Table S2-1).

### Estimation of Non-synonymous and Synonymous Rate (*K*_a_*/K*_s_)

The list of maize and rice orthologous genes was obtained from a previous study (Wang et al., 2014) to investigate the evolution rates between rice and maize. The protein sequences of GSR genes were used for a BLASTP search against the EnsemblPlants database^5^ in order to retrieve orthologous sequences from *Brachypodium distachyon*. All protein sequences were aligned using ClustalW with default parameters and back-translated to their corresponding DNA sequences by PAL2NAL. *K*_a_*/K*_s_ values were calculated by the maximum likelihood method implemented in KaKs_Calculator (Zhang et al., 2006). The significance of the difference in *K*_a_*/K*_s_ ratios between gene sets was evaluated by Kruskal–Wallis test.

### Combined Rhizotoxic Stresses for Validating Candidate Biomarkers

Six-days-old rice seedlings were exposed to five different combinations of metals/metalloid ions, each condition consisting 4 out of 5 metals/metalloid salts, namely 5 μM CuCl_2_, 25 μM CdCl_2_, 25 μM HgCl_2_, 50 μM K_2_CrO_4_, and 25 μM Na_2_AsO_4_⋅7H_2_O. Total RNA from rice root was then extracted and quantitative RT-PCR (qRT-PCR) was performed to analyze relative expression level of selected candidate biomarkers compared to water treated samples.

### Preparation of Total RNA and qRT-PCR Analysis

Total RNA was isolated from stress treated rice plants as described in plant materials section. Root sample (100 mg) was harvested and total RNA was isolated by use of Plant Total RNA Purification Kit (GeneMark, Taichung, Taiwan) according to the manufacturer’s instructions. RNA samples from 1 to 3 h were pooled together. 1 μl DNAase (GeneMark, Taichung, Taiwan) was used to eliminate possible DNA contamination. The concentration of total RNA sample was measured using NanoDrop ND2000 (NanoDrop Technologies, Wilmington, DE, United States). The cDNA was reverse transcribed from 1 μg RNA according to the manufacture’s recommendation. (Promega, Madison, WI, United States). Gene expression was determined by quantitative RT-PCR (Applied Biosystems, Foster City, CA, United States) using GoTaq^®^ qPCR Master Mix (Promega, Madison, WI, United States). The reaction was incubated at 95°C for 10 min, followed by 40 cycles of 95°C for 15 s, 60°C for 1 min. Gene-specific primers were designed and analyzed using PRIMER EXPRESs 3.0 Software (Applied Biosystems, Carlsbad, CA, United States). α-tubline (LOC_Os03g51600) was used as an internal control for normalization. Following PCR, a melting curve analysis was carried out. Relative quantification of specific mRNA levels was analyzed using the comparative CT method (ΔΔCt). All reactions were performed in triplicate reactions from three biological replicates. Primer sequences are listed in Supplementary Table S1.

### Gene Ontology (GO) Analysis

GOslim annotation file of rice genes was downloaded from Rice Genome Annotation Project^6^. The file format was then adjusted according to instructions on BINGO website^7^ to make a customized annotation file. Gene ontology analysis was carried out by BINGO^8^ using default settings with built-in GOslim ontology file and a customized annotation file for rice.

### Functional Gene Network Analysis

RiceNet v2 was used to create a probabilistic gene network of GSR genes and uniquely up-regulated genes^9^. All GSR genes were used as queries to construct the network. Uniquely up-regulated genes and stress-induced TFs and Kinases were used to build a probabilistic gene network for uniquely up-regulated genes. All networks were created using option I “gene prioritization based on network direct neighborhood” provided on RiceNet v2 web server. The obtained interaction results were than visualized by Cytoscape (Version: 3.2.1).

### Analysis of Gene Architecture and *Cis*-Regulatory Elements

The GFF3 file (Version 7.0) was downloaded from Rice Genome Annotation Project^10^. Excel was used to extract features annotated in the GFF3 file, including intron numbers and intron size. Statistical significance of the differences between percentages of intronless genes of different groups was calculated using 2-sample *z*-test. Kruskal–Wallis test and Dunn’s multiple comparisons test were used for statistical analysis when comparing average total intron length and total intron number of GSR genes, background genes and low-regulated genes. Mann–Whitney test was used to assess the significance for the difference of total intron length between top and bottom 50% of GSR genes.

### *Cis-Elements* Analysis

General stress response genes were queried against Elements^11^ and promoter sequences (1 kb upstream) were downloaded. MEME^12^ was used to detect enriched *cis*-elements in the 1000 bp upstream regions of GSR genes using the following command:

meme [sequence_file.fasta] -minw 6 -maxw 7 -dna -mod zoops -nmotifs 10 -maxsize 500000

PLACE^13^ and TOMTOM^14^ were used to match *cis*-elements found by MEME to known motifs. In addition, the number of known TFBSs of GSR genes were retrieved using Osiris promoter database^15^. The significance of predicted sites in GSR genes were compared to background genes using the FindMotif application^16^ and a custom-made Perl script. Statistical analysis of motifs involved a permutation test. Enrichment was defined by *p* < 0.05.

### Detection of ROS Levels in Rice Roots

Rice roots were labeled with 10 μM 5-(and-6)-chlormethyl-20,70-dichlordi-hydrofluorescein diacetate, acetyl ester (CM-H2DCF-DA) (Invitrogen, Carlsbad, CA, United States) for 30 min, then treated with 25 μM CdCl_2_ in the presence or absence of 5 μM DL-malic acid (Sigma, St. Louis, MO, United States) for 3 h. Fluorescence images of rice root were taken using Canon EOS500D (Canon, Tokyo, Japan) attached to a fluorescent microscope (Leica, Wetzlar, Germany) equipped with green fluorescent filter (excitation 450–490 nm, emission 500–530 nm). Fluorescence intensity was then quantitated by ImageJ^17^. All experiments were repeated at least three times. Statistical analyses of significant difference was carried out by Student’s *t*-test.

### Measurement of Malate Content in Rice Roots

Rice seedlings were treated with 25 μM CdCl_2_ for 3, 24, and 72 h. Root and shoot samples were then harvested and the malate level were analyzed using a malate assay kit (Biovision, Milpitas, Milpitas, CA, United States). All experiments were repeated for three times. Statistical analyses of significant difference between control and cadmium-treated samples was carried out by Student’s *t*-test.

### Cell Death Assay

Trypan blue staining is used to detect cell death as described by Duan et al. (2010) Rice seedlings were treated with 100 μM CdCl_2_ for 6 h with or without 10 μM of DL-malic acid, and then stained with trypan blue solution in a final concentration of 10 mg ml^-1^ for 15 min at room temperature. Seedlings were then rinsed with distilled water thoroughly for two times and submerged in water overnight. Images of rice root were taken by Canon EOS500D (Canon, Tokyo, Japan), the data was then quantitated using ImageJ. Three biological replicates were performed and statistical analyses of significant difference were carried out by Student’s *t*-test.

## Results

### General Stress Response (GSR) Genes in Rice

**FIGURE 1:**
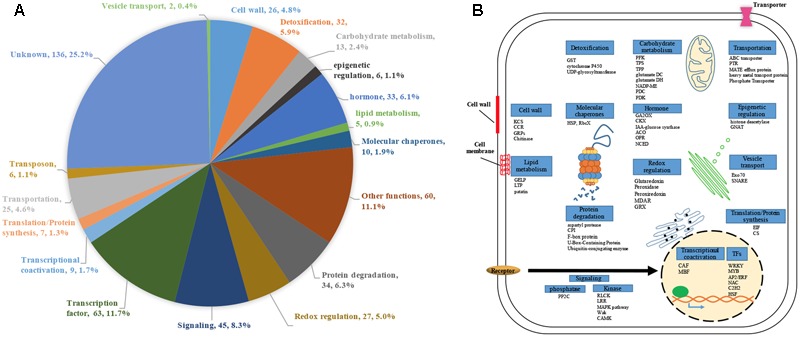
Functional classification of 539 GSR genes. Genes that were up-regulated (fold change ≥ 2, *p* < 0.05) under all eight rhizotoxins used in this study were termed GSR genes. **(A)** Pie charts representing different proportion of each category in GSR genes. For each category, category name, number of GSR genes in the category and percentage of total GSR genes were shown. Each gene was assigned to a specific functional category based on information from the following sources: GOslim terms, MapMan, gene description in the database of Rice Genome Annotation Project, and literatures. **(B)** Schematic diagram of a plant cell showing the representative genes under each GSR category. Genes are identified by abbreviation names. ACO, 1-Aminocyclopropane-1-carboxylic acid oxidase; CAF, CCR4-associated factor; CCR, cinnamoyl-CoA reductase; CKX, cytokinin dehydrogenase; CPI, Chymotrypsin protease inhibitor; CS, cysteine synthase; EIF, eukaryotic translation initiation factor; GA2OX, gibberellin 2-beta-dioxygenase; GELP, GDSL esterase/lipase protein; glutamate DC, glutamate decarboxylase; glutamate DH, glutamate dehydrogenase; GRPs, Glycine-rich proteins; GNAT, GCN5-related *N*-acetyltransferase; GST, glutathione-*S*-transferase; HSPs, heat shock proteins; KCS, 3-ketoacyl-CoA synthase; LTP, lipid transfer protein; MBF, multiprotein bridging factor; MDAR, monodehydroascorbate reductase; NADP-ME, NADP-dependent malic enzyme; NCED, 9-*cis*-epoxycarotenoid dioxygenase; OPR, 12-oxophytodienoate reductase; PDC, pyruvate decarboxylase; PDK, Pyruvate dehydrogenase kinase; PFK, 6-phosphofructokinase; RbcX, Chaperonin-like RbcX family protein; TFs, transcription factors; TPP, trehalose-6-phosphate phosphatase; TPS, trehalose-6-phosphate synthase; PTR, peptide transporter; PP2C, protein phosphatase 2C.

We performed genome-scale expression analysis using eight published microarray data generated in our lab (see Materials and Methods section). All transcriptomes were generated using identical growth conditions and protocols. Rice seedlings were submerged to six metal/metalloid stresses [5 μM CuCl_2_ (copper, Cu), 25 μM CdCl_2_ (cadmium, Cd), 25 μM HgCl_2_ (mercury, Hg), 50 μM K_2_CrO_4_ (chromate, Cr), 25 μM Na_2_AsO_4_⋅7H_2_O (arsenic, As(V)), 1 mM Na_3_VO_4_ (vanadate, V)], and two allelochemicals [50 ppm Ferulic acid (FA) and 10 μM juglone]. To understand the early response to rhizoroxins in rice, total RNA was extracted after 1 or 3 h treatment. Under these conditions, rice seedlings exhibited similar toxic effects: root growth was reduced to 52–79% of distilled water-treated control after 3 days exposure (Supplementary Figure S1). There were 8737 differentially expressed genes (DEGs) with fold change (FC) value greater than two under at least one stress treatment. Among these DEGs, four sets of genes were defined based on their expression patter across eight rihizotoxin treatments: (1) 539 general stress response genes (GSR) that were up-regulated under all stress conditions; (2) 591 uniquely regulated genes (unique-up or unique-down) with |FC|≥ 2 under one stress treatment and |FC|≤ 1.4 under the others; (3) 530 background genes with |FC|≤ 1.2 under all eight stress treatments; (4) 66 low-regulated genes (low-regulated) with 2 > FC > 1.2 under all eight stress treatments (**Table 1**). GOslim analysis was performed by using BINGO to determine over-represented functional categories in GSR genes. Enriched GOslim terms in GSR genes included transport, response to stress, lipid metabolic process, carbohydrate metabolic process and mitochondrion (Supplementary Figure S2). In order to gain more insight into detailed functional categories, GSR genes were manually classified into 19 categories based on information from following resources: GOslim data from BINGO, gene classification from MapMan software, and the putative function of each gene by literature search (**Figure 1A**). Representative genes in each category were listed and integrated in a schematic diagram representing a plant cell (**Figure 1B**). Around 25% of GSR genes were assigned to unknown category because of their unknown function. Genes encoding protein phosphatases and kinases were grouped into signaling category, making 8.3% of the total GSR genes. Among 28 kinases found in GSR genes, the most significantly enriched kinase family was the receptor-like cytoplasmic kinases (*RLCK*) (Supplementary Figure S3A). One tenth of GSR genes were TFs, and significantly enriched TF families included *WRKY, AP2/ERF, MYB, C2H2, NAC*, and *WOX* (Supplementary Figure S3B). The protein degradation category (6.3%) included genes encoding proteases and their inhibitors as well as those involved in the ubiquitin-proteasome system. The redox regulation category (5.0%) was formed of genes encoding antioxidant enzymes such as glutaredoxin, peroxidase, peroxiredoxin, and monodehydroascorbate reductase. Genes encoding cell wall proteins (e.g., Glycine-rich proteins) and important cell wall modifying enzymes (e.g., 3-ketoacyl-CoA synthase) were grouped to form the cell wall category (4.8%). The hormone category (6.1%) included important genes involving in hormone metabolism. For example, 12-oxophytodienoate reductase, 1-aminocyclopropane-1-carboxylate synthase, and 9-*cis*-epoxycarotenoid dioxygenase are responsible for biosynthesis of jasmonic acid, ethylene, and ABA, respectively, while gibberellin 2-beta-dioxygenase, cytokinin dehydrogenase and IAA-glucose synthase are related to deactivation or degradation of GA, cytokinin and IAA, respectively. All transporters were in the transportation category (4.6%). Genes involving in glycolysis, TCA cycle, GABA metabolism and trehalose biosynthesis were included in the carbohydrate metabolism category (2.4%).

**Table 1:** Number of differentially expressed genes in rice under eight rhizotoxic stresses.

		Up-	Down-	^b^Uniquely	Uniquely	Uniquely	^c^Generally	Generally	Generally	^d^Background	^e^Low-
Rhizotoxin	^a^DEGs	regulated	regulated	regulated	up	down	regulated	up (GSR)	down	genes	regulated
											
Cu	2666	1768	898	22	12	10	577	539	38	530	66
As(V)	4675	2527	2148	340	64	276	577	539	38	530	66
Cd	2577	1836	741	39	17	20	577	539	38	530	66
Hg	4029	2101	1928	53	31	22	577	539	38	530	66
Cr	2498	1801	688	16	9	7	577	539	38	530	66
V	3869	2486	1383	75	62	13	577	539	38	530	66
FA	2476	1742	734	23	18	5	577	539	38	530	66
Juglone	3033	2055	978	23	6	17	577	539	38	530	66
Sum (non- redundant)	8737	4696	4476	591	219	370	577	539	38	530	66

*^a^Differentially expressed genes, *|FC|*≥ 2. ^b^Differentially expressed genes with *|FC|*≥ 2 under one stress and *|FC|*≤ 1.4 under the other stress treatments. ^c^Differentially expressed genes with *|FC|*≥ 2 under all eight stress treatments. ^d^Genes with *|FC|*≤ 1.2 under all eight stress treatments. ^e^Genes with 2 > FC > 1.2 under all eight stress treatments.*

Recent transcriptome analysis in rice seedling treated with drought and salt has been investigated by Zhang et al. (2016). Comparison of the GSR genes with the genes up-regulated in seedlings treated with drought and salt stress shown highly overlapping. Although the genetic background of our study (TN67) is different from the previous study (N22), 35 and 39% GSR genes were highly expressed in N22 under drought and salt stress, respectively (Supplementary Table S3). Among the significantly expressed GSR genes, 50% of genes in the transcription factor category were up-regulated in N22 under drought stress, and up to 90% of genes in the molecular chaperones category were up-regulated in N22 under drought stress. The common stress-responsive transcripts identified here suggested that plants have evolved a mechanism of transcriptional regulation, which regulates the expression of regulatory genes under stress conditions.

A total of 24 genes related to cell wall, hormone and carbohydrate metabolism were selected for validation of microarray data by qRT-PCR (Supplementary Figure S4). Detailed information of genes in each category can be found in Supplementary Table S2-1.

### Genes Related to Transcriptional Coactivation Show Higher Rates of Evolution Than Other Genes

**FIGURE 2:**
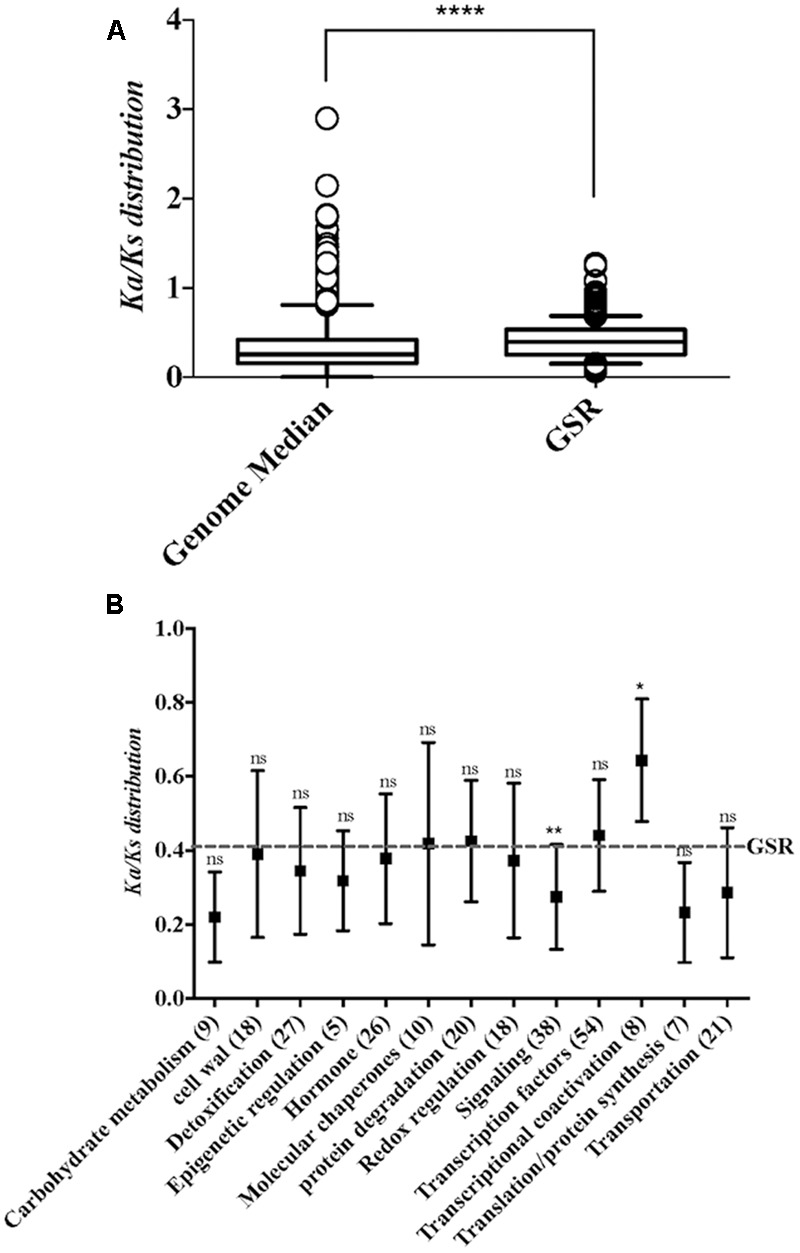
The distributions of *K*_a_*/K*_s_ ratio in whole genome and functional categories within GSR genes. **(A)** Boxplot depicts the *K*_a_*/K*_s_ ratio of the genome median and GSR genes. Because the data failed normality test, statistical significance was calculated using Mann–Whitney test. ^∗∗∗∗^*p* < 0.0001. **(B)** Boxplot represents the *K*_a_*/K*_s_ ratio of different functional categories in GSR genes. The *K*_a_*/K*_s_ mean value of GSR genes is represented by dotted line. The *x*-axis shows the functional categories and gene numbers in each category. Significance was calculated by comparing each category to total GSR genes. Because the data failed normality test, statistical significance between two groups was calculated using Kruskal–Wallis test followed by Dunn’s test for *post hoc* analysis. ^∗^*p* < 0.05, ^∗∗^*p* < 0.01; ns, not significant.

To investigate the conserved mechanism of GSR genes in plants, the non-synonymous and synonymous substitution ratio (*K*_a_*/K*_s_) between rice and maize orthologs were estimated by using the *K*_a_*K*_s_ calculator (Zhang et al., 2006). Nucleotide substitutions in protein-coding regions are divided into two classes, ones that change amino acid (non-synonymous) and those that do not (silent or synonymous). From these two numbers and the numbers of synonymous and non-synonymous sites, one can calculate two normalized values, *K*_a_, the number of non-synonymous substitutions per non-synonymous site, and *K*_s_, the number of synonymous substitutions per synonymous site. The list of maize and rice orthologs was obtained from published data (Wang et al., 2014). We found that GSR genes show a higher *K*_a_*/K*_s_ ratio than the whole genome (**Figure 2A**). To examine how selection pressures differ between GSR genes with different functions, we calculated *K*_a_*/K*_s_ ratios for different functional categories of GSR gene (**Figure 2B**). Of note, the *K*_a_*/K*_s_ ratios was significantly higher for the genes in transcriptional coactivation category than other GSR gene sets (Kruskal–Wallis test, *p* < 0.05), which suggests a faster rate of evolution for genes related to transcriptional coactivation.

### Functional Gene Network of GSR Genes

**FIGURE 3:**
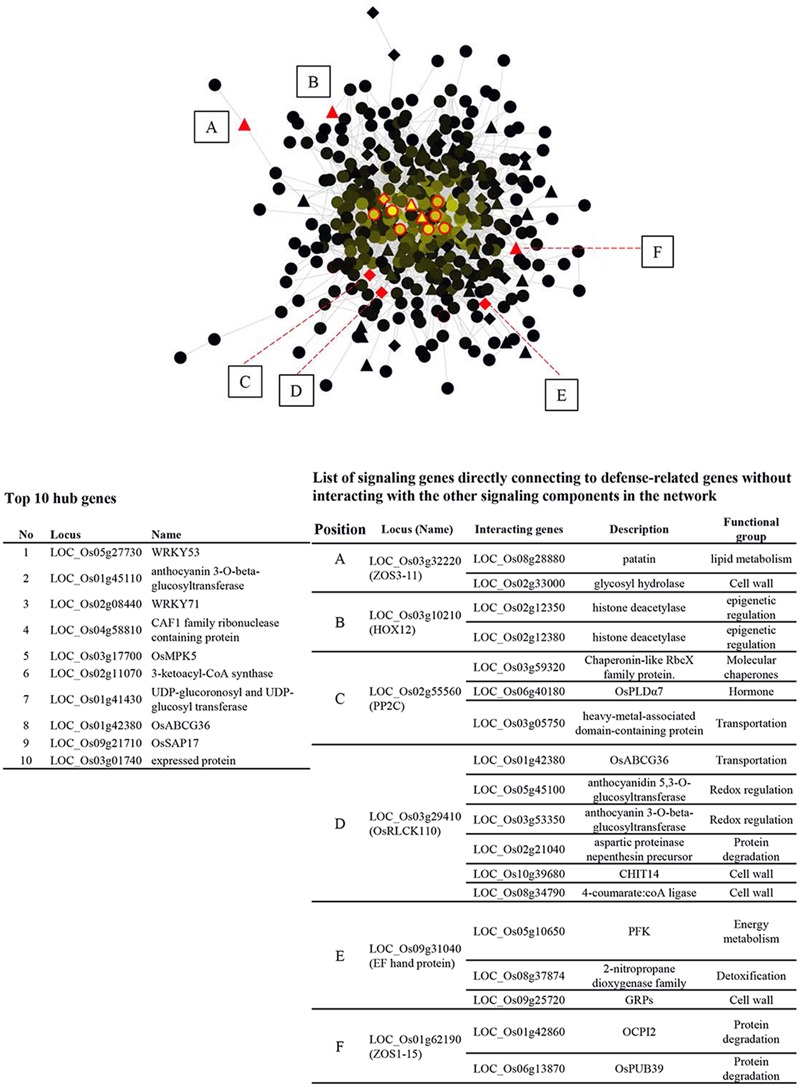
Regulatory network of GSR genes in rice. GSR genes were queried against RiceNet v2 and the data retrieved were imported into Cytosape (V3.2.1) to generate the regulatory network. Brightness of a node is proportional to the number of connection to other nodes. TFs are represented by triangles, while signaling genes are represented by diamonds. The other genes are represented by circles. The top ten hub nodes (most connected) in GSR regulatory network were shown in red edges and were listed in the lower left panel. Solid red triangles or diamonds (TFs or signaling genes) marked from A to E lack connection to other TFs or signaling genes and are connected to defense-related genes directly. These genes and their interacting partners were listed in the lower right panel.

In order to gain more insight into the interplay between signaling components and other functional genes, GSR genes were queried against RiceNet V2 to construct a probabilistic interaction network of GSR genes. The network contains 426 nodes and 2452 edges, demonstrating complicated connections between nodes (**Figure 3**). Nodes with a high degree of connection to others are hubs of the network. The top 10 hubs in the GSR network were *OsWRKY53*, anthocyanin 3-*O*-beta-glucosyltransferase, *OsWRKY71*, CAF1 family ribonuclease containing protein, *OsMPK5*, 3-ketoacyl-CoA synthase (KCS), UDP-glucoronosyl and UDP-glucosyltransferase, *OsABCG36, OsSAP17* and an expressed protein with unknown function (**Figure 3**).

Additionally, we found 3 TFs and 3 signaling genes connected to defense related genes without linking to the other signaling components in GSR network. The six nodes were *OsZOS3-11, OsHOX12, OsPP2C, OsRLCK110*, EF hand protein, and *OsZOS1-15* and their connected genes were listed in the right panel of **Figure 3**. To better understand how genes in different categories interact with signaling components, nodes of the same category were grouped to form a super node while signaling genes were grouped based on gene families (Supplementary Figure S5). Transportation category in GSR genes had the most connections with TFs and signaling genes though this category was 4.6% of GSR genes, which was smaller than categories such as protein degradation (6.3%) or hormone (6.1) (**Figure 1A** and Supplementary Figure S5). In addition, genes related to vesicle transport, namely *Exo70* and *SNARE*, were only connected to signaling genes (Supplementary Figure S5).

### Over-Represented *Cis*-Elements in GSR Genes

Regulatory motifs in promoter region are crucial for assembling the transcription machinery. Osiris^18^ was used to analyze the number of known TF-binding sites in the 1 Kb upstream promoter sequences of GSR genes. The average number of *cis*-elements in promoter regions was slightly but significantly higher in GSR genes compared to background genes (Supplementary Figure S6). For motif enrichment analysis, MEME was used to discover enriched *cis*-elements in GSR genes in the 1 Kb upstream promoter regions. Retrieved sequences were then compared to known motifs available in PLACE and TOMTOM databases. In addition, motifs known to be involved in stress response such as W-box were searched within 1 Kb promoter regions of GSR genes and background genes. The significantly overrepresented *cis*-elements in the promoter region of GSR genes were reported in **Table 2**. Motif enrichment analysis identified a *cis*-element SCGCGCS representing a CGCG box in GSR genes. Further analysis revealed that SCGCGCS was also enriched in genes that were induced transiently under heavy metal or phytochemical stress (Supplementary Figure S7). Additionally, three known regulatory motifs related to stress response, namely W-box, ABRE-like and RWRE, were also significantly enriched in GSR genes.

**Table 2:** Significantly enriched motifs in the 1 Kb upstream promoter region of GSRs.

^a^Motif	^b^In	^c^In background		^e^Matched
sequence	GSRs (%)	genes (%)	^d^*p*-value	motif
TTGACY	51.54	31.38	0	W-box
BACGTGKM	19.71	13.36	0.0146	ABRE-like
CGCGTT	12.53	6.07	0.0004	RWRE
SCGCGCS	31.83	21.86	0	CGCG box

*^a^Enriched motif sequences found by MEME using 1 Kb upstream promoter region of GSRs. ^b^Percentage of GSRs harboring the enriched motif. ^c^Percentage of background genes harboring the enriched motif. ^d^*p*-value after permutation test. ^e^Known motifs from PLACE and TOMTOM matching enriched motif sequences.*

### GSR Genes Are More Compact Compared to Background Genes

**FIGURE 4:**
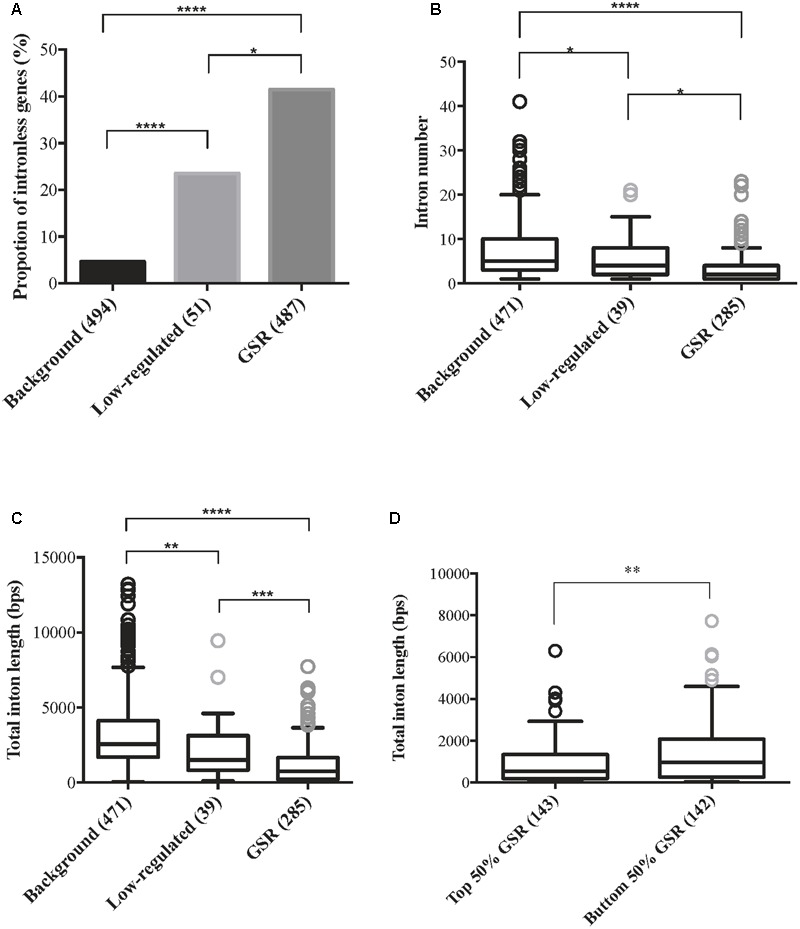
General stress response genes are more compact in gene architecture compared to non- or low- regulated genes. Genes were classified into three categories based on their expression level: background genes, 1.2 ≥ FC ≤ -1.2; low-regulated, 2 > FC > 1.2; GSR, FC ≥ 2 under all eight treatments. Data of intron number and intron length of all genes were retrieved from Rice Genome Annotation Project (http://rice.plantbiology.msu.edu/), therefore genes that were not assigned an LOC locus were discarded. Number of genes in each group was indicated in brackets. **(A)** Percentage of intronless genes within background genes, low-regulated genes and GSR genes. Statistical significance between two groups was calculated using 2-sample *z*-test. ^∗^*p* < 0.01, ^∗∗∗∗^*p* < 0.0001. **(B)** Boxplot represents the average intron number of background genes, low-regulated genes and GSR genes. Intronless genes were excluded in this analysis. Because the data failed normality test, statistical significance between two groups was calculated using Kruskal–Wallis test followed by Dunn’s test for *post hoc* analysis. ^∗^*p* < 0.05, ^∗∗∗∗^*p* < 0.0001; ns, not significant. **(C)** Boxplot represents the average total intron length of background genes, low-regulated genes and GSR genes. Intronless genes were excluded in this analysis. Because the data failed normality test, statistical significance between two groups was calculated using Kruskal–Wallis test followed by Dunn’s test for *post hoc* analysis. ^∗∗^*p* < 0.01, ^∗∗∗^*p* < 0.001, ^∗∗∗∗^*p* < 0.0001. **(D)** Boxplot represents the total intron length of top 50% and bottom 50% GSR genes. GSR genes were sorted by their FC value from lowest to highest. Intronless genes were excluded in this analysis. The average total intron length of the top 50% and bottom 50% genes were calculated. Because the data failed normality test, statistical significance was calculated using Mann–Whitney test. ^∗∗^*p* < 0.01.

Gene size is an important feature when studying gene expression and the presence of introns affects gene size. Gene architecture information such as intron number and intron length was retrieved from Rice Genome Annotation Project and genes without corresponding MSU locus were excluded. Therefore, there were 487 GSR genes, 51 low-regulated genes and 494 background genes subjected to downstream analysis. Because both introns and exons contribute to the size of a gene, the proportions of genes without introns in each group were calculated. A significantly higher proportion of intronless genes were discovered in GSR genes in comparison to low-regulated or background genes (**Figure 4A**). For genes harboring introns, there were significantly lesser number of introns in GSR genes compared to background genes (**Figure 4B**). Moreover, the average total intron length of GSR genes was significantly shorter than low-regulated genes or background genes (**Figure 4C**). These features seem to well correlated with the high, medium, and low FC value of GSR, low regulated and background genes, respectively (GSR, FC ≥ 2; low-regulated, 2 > FC > 1.2; background genes, FC ≤ 1.2). To understand if higher fold change value is correlated to more compact gene architecture, GSR genes were sorted according to FC value and then divided into two groups: top 50% and bottom 50%. Average total intron length of the top 50% GSR genes was significantly shorter than the bottom 50%.GSR genes (**Figure 4D**). However, when analyzing basal expression value of the three groups of genes, by utilizing the signal intensity from raw data after normalization of eight microarrays, we found that background genes showed significantly higher basal expression value than low-regulated and GSR genes (Supplementary Figure S8).

### Characteristics of Genes Uniquely Regulated by Individual Stressor

To reveal the uniquely regulated pathway by a specific stress, the MapMan software was used to identify specific biological processes (Thimm et al., 2004). Among the uniquely up-regulated genes under As treatment, we identified two functional enrichments (MapMan bins), including “Cell.organisation” (3 genes, *p* = 0.0347) and “RNA.regulation of transcription” (2 genes, *p* = 0.0448) (Supplementary Table S4). Among the uniquely down-regulated genes under As treatment, nine functional enrichments were found, including “RNA.regulation of transcription” (20 genes, *p* = 0.000035), “misc.cytochrome P450” (11 genes, *p* = 0.0335) and “misc.peroxidases” (5 genes, *p* = 0.016). The MapMan analysis highlighted the As-specific down-regulated genes, including cytochrome P450, peroxidase and transcription factor families (C2C2-Dof, C2H2, HB, and HSF), which might reveal the potential mechanism for the As toxicity. There were no significant functional enrichments under other stress conditions.

**FIGURE 5:**
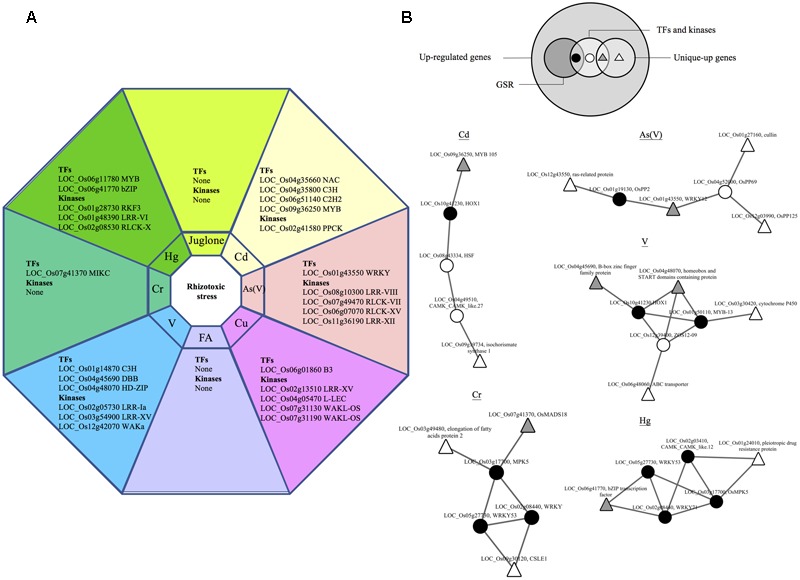
Hubs in GSR network connect unique-up TFs and defense genes. Genes that were up-regulated under only one out of eight treatment in this study were termed unique-up genes. **(A)** List of unique-up TFs and kinases under different rhizotoxic stress. **(B)** Functional networks of unique-up genes in rice. The Venn diagram shows the relation within up-regulated genes, GSR and unique-up genes. Large white circles represent two sets of genes used to construct regulatory network for Cd, Cr, As(V), Hg, and V. The first set consists of unique-up genes and the second set contains up-regulated TFs and kinases by the indicated rhizotoxin. TFs and kinases in the first set were shown in gray triangle while the others were shown in white triangle. For genes in the second set, GSR genes were represented by black circles while other genes were represented in white circles.

Signaling genes that were uniquely up-regulated in rice by one of the eight rhizotoxin treatments were discovered, including 12 TFs and 15 kinases (**Figure 5A**). To understand the possible interaction among these TFs, kinases along with the other uniquely up-regulated genes (Supplementary Table S2-2), RiceNetV2 was used to construct probabilistic interaction networks. The first attempt of using only unique-up genes to construct regulatory networks failed. However, when all up-regulated signaling genes were included together with unique-up genes, we were able to construct five regulatory networks and found the link from unique-up signaling components to unique-up defense-related genes (**Figure 5B**). Of note, in all cases, signaling components of GSR genes were shown to connect unique-up signaling components to unique-up defense-related genes.

### OsPPCK2 Is a Candidate Cadmium Biomarker

**FIGURE 6:**
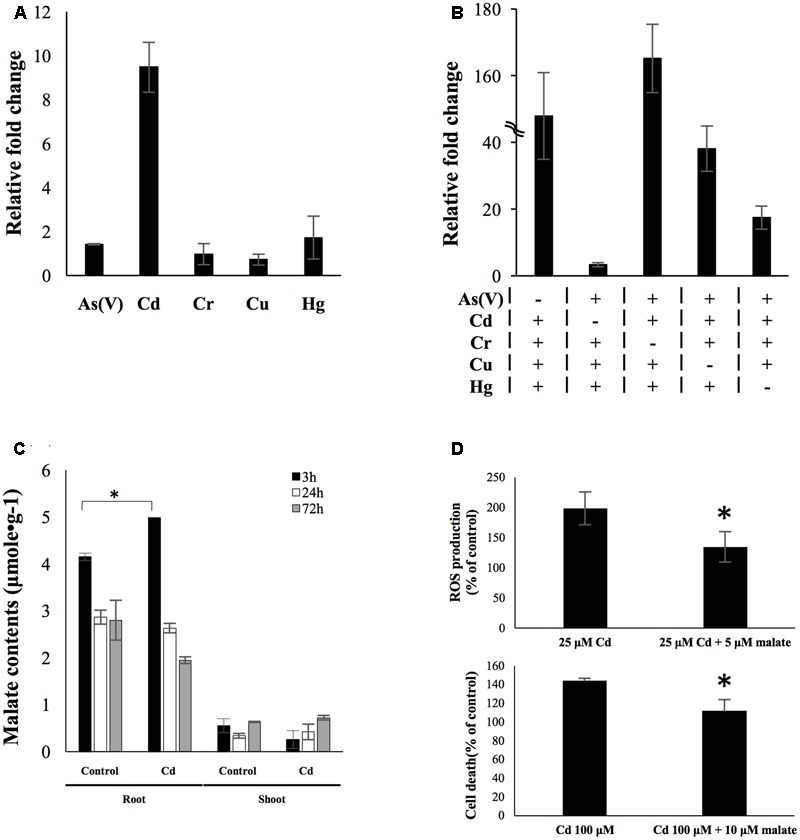
*OsPPCK2* as a candidate biomarker and its role in defending cadmium stress. **(A)** Expression level of *OsPPCK2* under individual metal/metalloid treatment. Quantitative RT-PCR was carried out to analyze the relative transcript abundance compared to water-treated sample. **(B)** Expression level of *OsPPCK2* under a variety of mixtures of four metals/metalloid stresses. Quantitative RT-PCR was carried out to analyze the relative transcript abundance compared to water-treated sample. A total of five combinations of metal stresses were tested, each containing four out of five metals/metalloid stresses, namely 25 μM As(V), 25 μM Cd, 25 μM Hg, 5 μM Cu, 50 μM Cr and 1 mM V. **(C)** Malate accumulation in rice root after 3 h exposure to 25 μM cadmium. **(D)** Exogenous applied malate reduced cadmium-induced ROS accumulation (upper panel) and cell death (lower panel) in rice root. Statistical analyses of significant difference were carried out by Student’s *t*-test. Asterisks indicate significance (*p* < 0.05).

A total number of 219 genes were identified as uniquely up-regulated by different rhizotoxin treatments (**Table 1**). Among these genes, we tried to find potential biomarkers which could be used to detect specific metal contamination. There were six metals/metalloid stresses in the present study [25 μM As(V), 25 μM Cd, 25 μM Hg, 5 μM Cu, 50 μM Cr, and 1 mM V]. We eliminated genes that were uniquely up-regulated under vanadate treatment because the high concentration of vanadate (1 mM) was unlikely to exist in environments. Among the uniquely up-regulated genes, *LOC_Os02g41580* was of special interest because qRT-PCR confirmed its low induction level by As, Cr, Cu, and Hg treatment for 3 h while a ninefold change was observed after cadmium treatment for 3 h (**Figure 6A**). To further investigate the role of *LOC_Os02g41580* in rice under cadmium stress, we analyzed its gene expression and discovered that the gene induction remained over sixfold after 72 h of cadmium treatment (Supplementary Figure S10).

To determine the expression pattern of this candidate biomarker under multiple metal stresses, we exposed rice seedlings to five stress conditions, each comprising four out of the five metals/metalloid [As(V), Cd, Hg, Cu, and Cr]. Expression level of *LOC_Os02g41580* was then determined by qRT-PCR. In the presence of cadmium in combination of three other metals/metalloid, expression level of LOC_Os02g41580 was found induced between 17- and 165-fold. In the presence of both Hg and Cd, the induction level was higher than the other combinations. However, in the absence of cadmium, LOC_Os02g41580 only exhibited 3.3-fold changes under a mixture of other metals/metalloid stress (**Figure 6B**). This result indicated that only cadmium will cause high transcriptional response of this transcript even under the presence of multiple heavy metals, while combinations of other heavy metal stresses will generate a distinctly moderate increase in the transcript abundance. We further identified LOC_Os02g41580 as phosphoenolpyruvate carboxylase kinase 2 (*OsPPCK2*) using protein blast (Supplementary Figure S9).

### Malate Reduced Cadmium Toxicity

Phosphoenolpyruvate carboxylase kinase regulates the activity of phosphoenolpyruvate carboxylase (PEPC) via phosphorylation (Fukayama et al., 2006). In rice, PEPC participates in the provision of malate (Masumoto et al., 2010). We then measured the malate content in rice roots and shoots after exposed to 25 μM cadmium for 3 and 72 h. Significant malate accumulation was observed in rice roots after 3 h treatment of cadmium, but at 72 h time point the malate content declined to a level similar to untreated rice roots (**Figure 6C**). The function of this early accumulation of malate was studied by applying exogenous malate accompanying cadmium stress to rice seedlings. The result showed that exogenous applied malate led to 64 and 32% reduction of cadmium-induced ROS accumulation and cell death (**Figure 6D**), respectively.

## Discussion

Due to their sessile nature, plants acclimatize to ever-changing environments by fine-tuning metabolic and signaling pathways thus forming responses unique to plants (Mustroph et al., 2010). By analyzing the rhizotoxin-responsive transcriptome sets in rice microarray data, we identified common GSR genes. Among these genes those with regulatory roles are of special interest and are discussed in the following context.

### Gene Architecture and Evolution of Rice GSR Genes

In this study, a motif SCGCGCS with sequence similarity to the CGCG box was significantly enriched in the promoter regions of GSR genes. The CGCG box is the core binding motif of CAMTA TFs (Benn et al., 2014). CAMTA TFs are involved in multiple stress signaling pathways and responses (Reddy et al., 2011). A simple model has been proposed that CAMTA TFs connect gene regulation and calcium signaling triggered upon exposure to stress (Doherty et al., 2009). Cellular Ca^2+^ is an important messenger in activating diverse responses to environmental cues and the majority of stress signals, biotic or abiotic alike, elicit changes in plant cellular Ca^2+^ level (Reddy et al., 2011). Altered cellular homeostasis of Ca^2+^ influences the activity of CAMTA TFs by post-translational modification through calmodulin proteins. The activated CAMTA TFs subsequently induce expression of stress-responsive genes such as *CBF2* (Doherty et al., 2009). In *Arabidopsis*, the Rapid Stress Response Element (RSRE; CGCGTT), which is the binding site of CAMTA3, was found to be sufficient to initiate a transient response to multiple biotic or abiotic stresses at early stage (Walley et al., 2007; Benn et al., 2014). Here we report that a significant proportion of GSR genes and transiently induced genes harbor SCGCGCS *cis*-elements (**Table 2** and Supplementary Figure S7), suggesting that in both *Arabidopsis* and rice, regulation of GSR by CAMTA TFs binding to CGCG box may be an evolutionarily conserved mechanism.

The relationship between exon–intron architecture and gene regulation has long been a topic of fundamental interest to biologists. Jeffares et al. (2008) discovered that rapidly regulated genes in response to stress tend to have fewer introns in *Arabidopsis* and proposed that selection against introns is critical for genes that require rapid regulation. Here we reveal that GSR genes, which are induced within 3 h of exposure to rhizotoxins, tend to be intronless compared to low-regulated or background genes, indicating that selection against introns in rapidly regulated genes may be a common feature in monocot and dicot plants. In addition, GSR genes with higher FC value were more compact (**Figure 4**). A transcript’s size is a determinant of time of transcription, given that most genes are transcribed at a similar rate (e.g., 3.5 Kb/min in human) (Fuchs et al., 2014). Therefore, a possible explanation to the negative correlation observed in this study is that transcripts with smaller total intron size may be produced more rapidly, as has been suggested for miRNAs (Hobert, 2008). Moreover, introns have long been known to function in enhancing gene expression, termed intron-mediated enhancement, although the mechanism is not fully understood (Gallegos and Rose, 2015). Indeed, background genes and low-regulated genes, which harbor longer total intron length than GSR genes, were shown to have higher basal expression level compared to GSR genes (Supplementary Figure S8). Genes with low basal expression are more likely to be highly induced upon environmental stimuli (Rubinstein et al., 2010). Taken together, total intron length of GSR genes may affect the efficiency of transcription and the basal expression, therefore further affects the magnitude of the gene induction under stressed conditions.

It has long been recognized that the protein evolution rate is influenced by its expression level. However, our understanding of evolution rate variation among stress regulated genes remains limited. A previous study reported that the rate of protein evolution is positively correlated with developmental timing of expression (Good and Nachman, 2005). Genes expressed late in spermatogenesis were found to evolve more quickly. In plants, Warren et al. (2010) found that defense response genes are under stronger diversifying selection. Recently, analysis of the different evolutionary rate of *Arabidopsis* hub genes under pathogen infection showed that rapidly evolving hub genes are likely to ensure successful defense (Jiang et al., 2016). Protein-encoding genes evolving at the neutral rate have *K*_a_*/K*_s_ ratios equal to 1 (Li et al., 1985). Most genes, however, are subject to strong purifying selection, resulting in lower non-synonymous relative to synonymous substitution rates (i.e., *K*_a_*/K*_s_ < 1). Here, our natural selection analysis between rice and maize orthologs showed greater *K*_a_*/K*_s_ ratio for genes related to transcriptional coactivation than other GSR gene categories and the genome median, which suggests that a more rapid evolutionary rate. We also assessed the *K*_a_*/K*_s_ ratios between rice and *Brachypodium distachyon* ortholgs, and genes related to transcriptional coactivation were also having higher *K*_a_*/K*_s_ ratios (Supplementary Figure S11). Previous study has shown that constitutive expression of the stress-response transcriptional coactivation protein would enhance the tolerance of transgenic plant to abiotic and biotic stresses (Suzuki et al., 2005). The higher rate of divergence among these genes may be due to their roles in controlling the plant transcriptional coactivation in response to ever changing environment.

### Hubs in GSR Network May Mediate Stress-Specific Response

Twenty percent of GSR genes discovered in this study were transcription factors or genes related to signal transduction (**Figure 1A**), which agrees with previous report on the alarm phase of plant stress responses (Kosová et al., 2011). These factors could shape the later response of organisms to environmental stimuli by interacting with downstream targets to form a transcription regulatory network (Yamaguchi-Shinozaki and Shinozaki, 2006). Highly connected genes, or hubs, in a network tend to play essential roles in biological processes and deletion of hub genes is more likely to be lethal (Jeong et al., 2001). Here we have identified *OsWRKY53, OsWRKY71*, and *OsMPK5* among the top 10 hub genes in the GSR network, suggesting their key roles in modulating early stress responses in rice (**Figure 3**). Overexpression of *OsWRKY53* or *OsWRKY71* enhances rice resistance to biotic stress while overexpressing *OsMAPK5* leads to increased tolerance to abiotic stress (Xiong and Yang, 2003; Chujo et al., 2007; Pandey and Somssich, 2009). However, transgenic plants that are resistant to one stress are often susceptible to another or accompanied with yield penalty because of extensive cross-talk between signaling pathways. In rice, *OsMAPK5* enhances abiotic stress tolerance but represses the plant defense response (Chen and Ronald, 2011). Cabello et al. (2014) proposed that it is possible to enhance stress resistance in crops without producing negative pleiotropic effects by intervening genes at the end of signal transduction cascade. In this study, we identified six nodes (TFs or signaling genes) in GSR network that directly connect to defense related genes without connection to other signaling components (**Figure 3**). For example, a protein phosphatase 2C (OsPP2C, LOC_Os02g55560) was found in GSR network connecting to a gene belonging to phospholipase D alpha protein family (LOC_Os06g40180), suggesting a signaling pathway in ABA regulation (Hirano et al., 2008). We suggest that these genes are potential targets for genetic engineering to increase stress tolerance in rice.

By applying a strict filtering criteria we identified 12 uniquely induced TFs, but there was no TFs unique to allelochemical stimuli, possibly because the response was similar to that of other abiotic stresses (**Figure 5A**) (Golisz et al., 2008). How unique up-regulated TFs may determine the specific transcriptional profile is an important question but in most cases it remains unexplored because the signaling specificity could be determined by many processes. One of the processes is cooperation between different TFs to activate particular genes (Vaahtera and Brosché, 2011). Using network analysis we found that uniquely up-regulated TFs connect to defense-related genes through non- uniquely regulated TFs, including hubs in GSR genes (**Figure 5B**). In the case of As(V), *OsWRKY12* was uniquely up-regulated and linked to a cullin family gene (LOC_Os01g27160) through a PP2C gene (LOC_Os04g52000) (**Figure 5B**). In rice, cullin OsCUL3a has been reported to negatively regulate cell death (Liu et al., 2017), which may also be important for the survival under As(V) stress. In addition, hubs in GSR genes seem to be involved in multiple stress-specific networks. For example, *WRKY53* was discovered in Hg and Cr stress networks (**Figure 5B**). Therefore, it is possible that some TFs in GSR genes are required for stress-specific response via cooperation with uniquely up-regulated TFs. Accumulating evidence supports the idea that extensive cross-talk exists in plant signaling pathways triggered by environmental stimuli, and TFs may be the key node for such cross-talk (Nakashima et al., 2014). Recently, transcriptional profiling coupling co-expression network analysis has successfully uncovered proteins that mediate cross-talk between abiotic and biotic stress (Sharma et al., 2013). Here we reported *WRKY53 and WRKY71*, two hubs in the GSR network, that seem to be involved in stress cross-talk, mediating responses unique to individual rhizotoxic stress possibly by connecting uniquely up-regulated TFs and defense genes.

### OsPPCK2 Is a Candidate Cadmium Marker and May Involve in Alleviating Cadmium Stress in Rice

Over the past 15 years, different biological methods have been developed to detect environmental contamination, especially heavy metal pollutants (Ernst et al., 2000; Kovalchuk et al., 2001; Bundy et al., 2007). However, to our knowledge there is no specific biomarker that is capable of detecting single metal contamination accurately in the environment without being biased by other soil contaminants. In this study, we assess stress specificity of candidate biomarkers under complex environments by treating rice with five different combinations of five metals/metalloid (**Figure 6B**). In the multiple stress experiment, we used the same concentration of each rhizotoxin as for single stress treatment to amplify common effects caused by rhizotoxins. Therefore, biomarkers that showed specificity to a particular stressor under multiple stress treatment, are unlikely to be due to common effects such as rapid accumulation of ROS caused by rhizotoxins. Under this condition, *OsPPCK2* was found to be highly induced only in the presence of cadmium (**Figure 6B**). Our result demonstrates that *OsPPCK2* is a strong candidate biomarker for early detection of cadmium contamination in soil. However, it needs to be noted that a single biomarker alone may not always provide a good assessment of heavy metal contamination.

Moreover, induction of *OsPPCK2* may benefit rice to cope with cadmium toxicity by elevating internal malate levels in root (**Figures 6C,D**). Organic acids such as malate, citrate, and oxalate are metal-chelating agents, which play import roles in mediating plant tolerance to a variety of metal toxicity in different plant species (Hall, 2002; Chen et al., 2015). In both C3 and C4 plants, PPCK activates PEPC that contributes to provision of malate (Masumoto et al., 2010). Here we revealed induction of *OsPPCK2* and accumulation of malate under cadmium stress in rice root. This specific regulation of *OsPPCK2* in response to cadmium may be a defense mechanism evolved in rice to contend with cadmium toxicity.

## Conclusion

The combined analysis of microarray data integrating different statistical methods allowed us to narrow down the differential expression genes to a set of GSR genes. The shared intronless architecture and *cis*-element motifs of GSR genes might provide clues for further in-depth molecular studies. Among the GSR genes identified in this study are interesting hub candidates such as WRKYs and MAPK, which were highly correlated with other GSR genes. Meanwhile, a powerful candidate biomarker (*OsPPCK2*) for specific detection of cadmium contamination was identified according to the submerged analysis of these metal stress microarray data.

## Author Contributions

L-YH and C-WL analyzed the data and wrote the manuscript. L-YH, C-WL, and R-HL contributed equally as first author. L-YH, C-YC, and Y-CW performed the RNA experiment and PCR analysis. C-WL and C-HC performed the *K*_a_/*K*_s_ calculation. L-YH, C-WL, and C-HC performed the network component analysis. R-HL and H-JH provided the technical assistance. H-JH organized this work. All authors contributed to and approved the final manuscript.

## Conflict of Interest Statement

The authors declare that the research was conducted in the absence of any commercial or financial relationships that could be construed as a potential conflict of interest.

## Footnotes

1 http://www.ncbi.nlm.nih.gov/geo/

2 http://apps.cytoscape.org/apps/bingo

3 http://mapman.gabipd.org/web/guest/home

4 http://rice.plantbiology.msu.edu/

5 http://plants.ensembl.org/Brachypodium_distachyon/Info/Index

6 http://rice.plantbiology.msu.edu/

7 http://www.psb.ugent.be/cbd/papers/BiNGO/Customize.html

8 http://apps.cytoscape.org/apps/BiNGO

9 http://www.inetbio.org/ricenet/

10 http://rice.plantbiology.msu.edu/pub/data/Eukaryotic_Projects/o_sativa/annotation_dbs/pseudomolecules/version_7.0/all.dir/all.gff3

11 http://element.mocklerlab.org

12 http://meme-suite.org

13 http://agrid.dna.affrc.go.jp

14 http://meme-suite.org/tools/tomtom

15 http://bioinformatics1.smb.wsu.edu/cgi-bin/Osiris/cgi/visualize_select.pl

16 http://hgdownload.soe.ucsc.edu/admin/exe/

17 http://imagej.nih.gov/ij/

18 http://bioinformatics1.smb.wsu.edu/cgi-bin/Osiris/cgi/visualize_select.pl
