# Supplementary material for: Integrating Early Transcriptomic Responses to Rhizotoxins in Rice (Oryza sativa. L.) Reveals Key Regulators and a Potential Early Biomarker of Cadmium Toxicity
Source: Front Plant Sci. 2017 Aug 18;8:1432. doi: 10.3389/fpls.2017.01432 (PMC5563368; doi:10.3389/fpls.2017.01432)
Supplement: Supplementary file 4 [file Data_Sheet_1.DOCX]

Supplementary Material

Integrating early transcriptomic responses to rhizotoxins in rice (*Oryza sativa. L.*) reveals key regulators and a potential early biomarker of cadmium toxicity

Li-Yao Huang^1,‡^, Chung-Wen Lin^1,‡^, Ruey-Hua Lee^2,‡^, Chih-Yun Chiang^1^, Yung-Chuan Wong^1^, Ching-Han Chang^1^, and Hao-Jen Huang^1,2,^*

*** Correspondence:** Hao-Jen Huang, haojen@mail.ncku.edu.tw

**Table S1.** Primer sequences used in qRT-PCR

| **Locus** |  | **Sequence (5'->3')** |
| --- | --- | --- |
| LOC_Os02g41670 | Forward primer | GAGGTGGTGGTTTGTTTCGAG |
|  | Reverse primer | TACTTTAGCCGCTTGCCAGG |
|  | Product length | 150 |
| LOC_Os04g43800 | Forward primer | TTGCTGAACAGAGGACCGAC |
|  | Reverse primer | AGTAGCTGCCTGCCTCTATG |
|  | Product length | 200 |
| LOC_Os03g13300 | Forward primer | CGTGCTAATTTGAGTGGAGCC |
|  | Reverse primer | CTCTCGGGAGATGAGTGCAG |
|  | Product length | 139 |
| LOC_Os04g45970 | Forward primer | CCATGCGCTTTAGGTGGAGT |
|  | Reverse primer | ATGGTCACTCCCTTCTTGGC |
|  | Product length | 131 |
| LOC_Os08g04800 | Forward primer | GAGGCGTACATCCACCTCGTC |
|  | Reverse primer | TTGCTGATGTAGAGCTGCTTGA |
|  | Product length | 128 |
| LOC_Os04g37430 | Forward primer | CGTGCCAAACAGCGTAACTA |
|  | Reverse primer | AGCAATCGACCTATCCGAAGA |
|  | Product length | 134 |
| LOC_Os05g05680 | Forward primer | ACATAACCGCTTTTGCTATTCAAGA |
|  | Reverse primer | ACTGACAAACCGTGACACACT |
|  | Product length | 138 |
| LOC_Os05g10650 | Forward primer | CTACCTCGAAGGCAAGGGC |
|  | Reverse primer | GATCCTTGATCTTGGCGCAC |
|  | Product length | 200 |
| LOC_Os04g48850 | Forward primer | CTTGCTCTGCTAATGCTGCG |
|  | Reverse primer | ATCACACGTACCTGCATCGG |
|  | Product length | 125 |
| LOC_Os02g48770 | Forward primer | TGTGTTGTTGGGGACCGATT |
|  | Reverse primer | ATTCGCGTGGGCTAGTTCAT |
|  | Product length | 148 |
| LOC_Os02g11070 | Forward primer | TGCTCCTTTGGAATTGGCAC |
|  | Reverse primer | CACACCCAAGCTGAAGTGGT |
|  | Product length | 177 |
| LOC_Os01g09570 | Forward primer | TCCAAGTGCAACGGGACAG |
|  | Reverse primer | AGGATGAAACCAGCCTTGCT |
|  | Product length | 196 |
| LOC_Os12g42280 | Forward primer | TTCAAGCTCGGCGAGATGT |
|  | Reverse primer | AGGTGGAAGCAGAAGCAGTC |
|  | Product length | 146 |
| LOC_Os01g71310 | Forward primer | CTCGTTATCGGTGTCTCGGG |
|  | Reverse primer | GTTCCAACCATTGTGCGTCC |
|  | Product length | 150 |
| LOC_Os08g17500 | Forward primer | TGCGTACGTGCCTGTATGTT |
|  | Reverse primer | AGTGAAACAGCACGCTATTTTTGA |
|  | Product length | 132 |
| LOC_Os08g02700 | Forward primer | TCTATGCTCTCGCAATGTCG |
|  | Reverse primer | AATCATCAGGTAGTACTCCACTTT |
|  | Product length | 154 |
| LOC_Os04g12720 | Forward primer | CAATGGCCTTTGCAACTCCG |
|  | Reverse primer | GCCATTAACAATTGCCTCCAGC |
|  | Product length | 148 |
| LOC_Os07g37730 | Forward primer | AGGAACAGGTTCTACGTGGC |
|  | Reverse primer | CCCTCTCTTCCAGAACGTGTG |
|  | Product length | 147 |
| LOC_Os07g13800 | Forward primer | TGGTTACTGCGAGGTACGTG |
|  | Reverse primer | CTCTGCTTAACCTCAGCCCC |
|  | Product length | 133 |
| LOC_Os02g51930 | Forward primer | TGGCCAAAGAGTCGTAGCTC |
|  | Reverse primer | ATAGGCAGTAGGCCAGTAGC |
|  | Product length | 123 |
| LOC_Os06g11290 | Forward primer | CAGGATCCCGTTGTTGGCT |
|  | Reverse primer | TTCATGCTTGTCCGTATCACAT |
|  | Product length | 159 |
| LOC_Os06g11210 | Forward primer | GTGTTAGTATATGCCTGCGTGC |
|  | Reverse primer | ATGTGGTGTGAATACATCTGCG |
|  | Product length | 145 |
| LOC_Os06g11200 | Forward primer | ATCAATAATGCAGGCACCCG |
|  | Reverse primer | TGCATGTCTTGATGTCTTCTCAC |
|  | Product length | 120 |
| LOC_Os01g55240 | Forward primer | CGGTACACCATGACCACGAG |
|  | Reverse primer | TCCGAAACAGGAAAGGAGCC |
|  | Product length | 182 |
| LOC_Os02g41580 | Forward primer | ATGGAGTTTACCCCCAAAATCAC |
|  | Reverse primer | GAGCGGTCGACGGACTTGA |
|  | Product length | 167 |
| LOC_Os03g51600 | Forward primer | TCGCAGCATCAACCCAATC |
|  | Reverse primer | GCAACCAGTCCTCACCTCAT |
|  | Product length | 272 |

**Table S2.** Expression data of GSR genes

*Note that Supplementary Table S2 is available in a separated Excel file named Supplementary Table.S2.xlsx*

**Table S3.** GSR genes in different rice cultivars under drought and salt stresses

*Note that Supplementary Table S3 is available in a separated Excel file named Supplementary Table.S3.xlsx*

**Table S4.** MapMan enrichment analysis for uniquely regulated genes

*Note that Supplementary Table S4 is available in a separated Excel file named Supplementary Table.S4.xlsx*

**Fig.** **S1.** Percentage of root length reduction compared to water-treated control after 3 days treatment. Six-days-old rice seedlings were exposed to different stresses including 5 μM CuCl2, 25μM CdCl2, 25μM HgCl2, 50μM K2CrO4, 25μM Na2AsO4•7H2O, 1 mM Na3VO4, 50 ppm FA and 10μM juglone for 3 days then the root length was measured. Control plants were treated with water in parallel for the indicated time. Data was collected from three biological replicates and presented as mean ± SD.


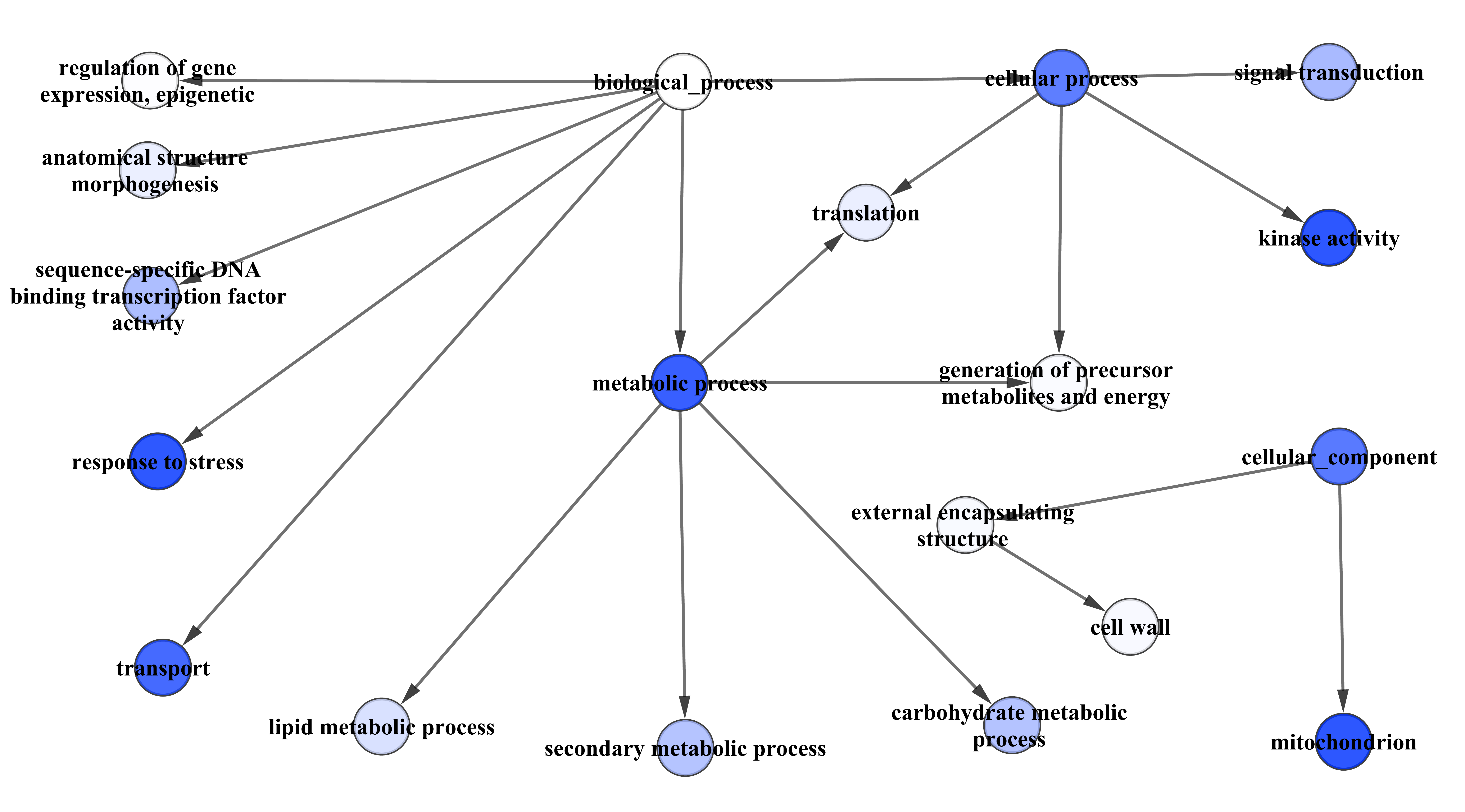


**Fig. S2.** Selected enriched GOslim categories in GSR genes. MSU loci (e.g. LOC_Os10g33000.1) of GSR genes were inputted into BiNGO, a Cytoscape plugin, and enriched GOslim terms were acquired using default settings with built-in GOslim ontology file and a customized annotation file for rice. The darker the node color means the more significant of enrichment of a GOslim term.

(A)

(B)


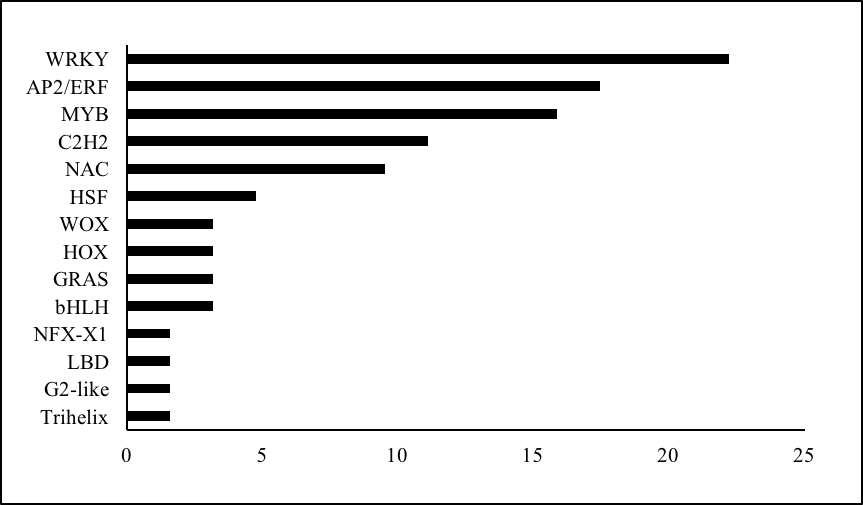


*

**Fig. S3.** Overrepresented protein kinase and transcription factor families in GSR genes. Kinases (A) and transcription factors (B) in GSR genes were grouped based on families and were represented in percentages relative to total number of kinases or transcription factors. Fisher’s exact test was used to assess the significance of overrepresented protein kinase and transcription factor families. Asterisks indicate significantly overrepresented families (p < 0.05).

**Fig. S4.** Validation of expression induction by individual metal/metaloid stresses for GSR genes in cell wall, hormone and carbohydrate metabolism categories. Rice roots were treated with water or eight different metal/metaloid stress separately. Total RNA was extracted and qRT-PCR was performed to measure gene induction level. Individual stress-treated samples were compared to water-treated sample. Data was shown as mean relative expression ± SD for three replicate qRT-PCR reactions from three independent samples. The maximum value of x-axis (fold change) was restricted to 10 for better interpretation of the figures.

**Fig. S5.** Functional network among signaling components and the other functional categories of GSR genes. Nodes in Fig. 3 with the same functional category were grouped to form a super node, while TFs and signaling genes were grouped based on gene families. Percentage of different types of interaction in each category is shown in pie charts. For pie chart and lines between super nodes, blue, red and green represent different interaction. Blue represents the interaction between kinases and 13 functional categories. Red lines represent the interaction between TFs and 13 functional categories. Green represents the interaction between TFs and kinases. Number of lines and size of circles are proportional to number of connections.

**Fig. S6.** GSRs harbored more cis-elements than background genes. Osiris was used to identify known cis-elements in the 1 Kb upstream promoter region. Data was presented as average number of cis-elements ± SD. Because the data failed normality test, statistical significance was calculated using Mann-Whitney test. ****p < 0.0001

**
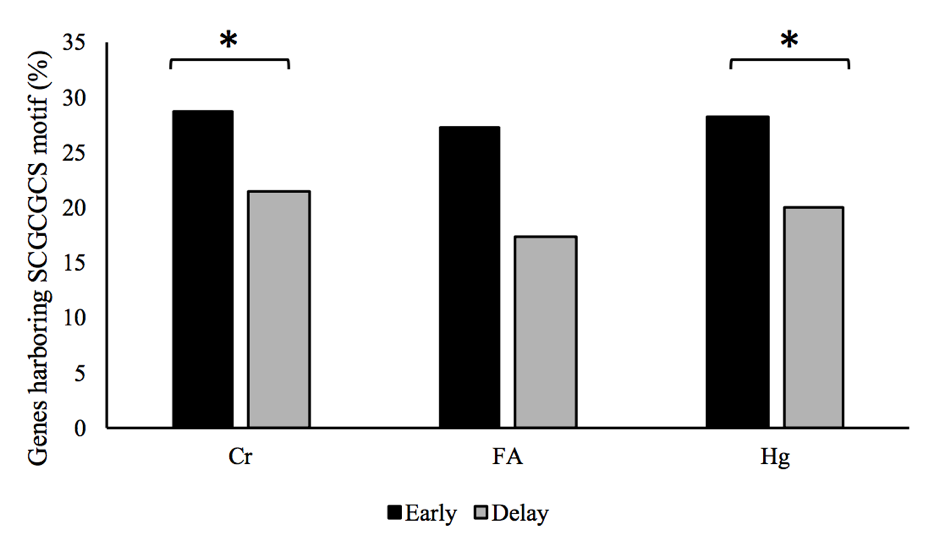
**

**Fig. S7.** SCGCGCS enrichment analysis in early and delay response genes under Cr, FA, and Hg treatment. The rice microarray data with short and long exposure of Cr, FA, and Hg treatment were downloaded from GEO database (GEO: GSE41733, GSE34899, and GSE41719). The induced genes were characterized as early (significantly upregulated at 1+3 h [i.e. >= 2-fold] and then regulated at 24 h [i.e. <= 1.4-fold]) and delay (significantly upregulated at 24 h only). The SCGCGCS motif enrichment analysis in early and delay response genes were compared to Cr, FA, and Hg short and long exposure induced genes, respectively. Statistical analysis of significant difference was carried out by permutation test. Asterisks indicate significance (p < 0.05).

**Fig. S8.** Average basal expression of background genes, low-regulated genes and GSR genes. Data are shown as mean basal expression value after normalization of eight microarray data sets ± SD. Because the data failed normality test, statistical significance between two groups was calculated using Kruskal-Wallis test followed by Dunn’s test for post-hoc analysis. **p < 0.01; ****p < 0.0001.

**
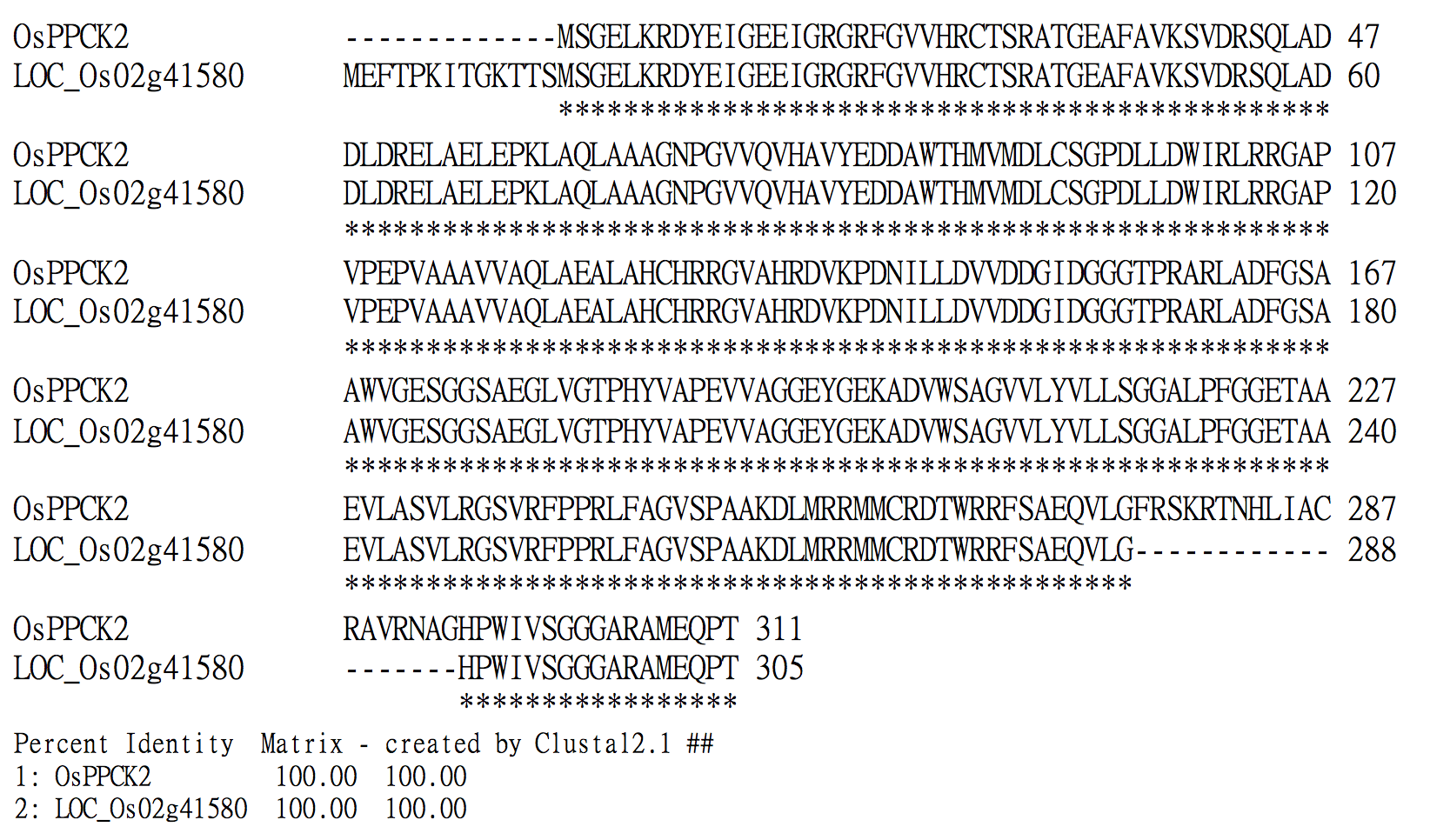
**

**Fig. S9.** Protein sequence alignment between LOC_Os02g41580 and OsPPCK2. ClustalW2 (<http://www.ebi.ac.uk/Tools/msa/clustalw2/>) was used to align protein sequences and measure percent identity.

**
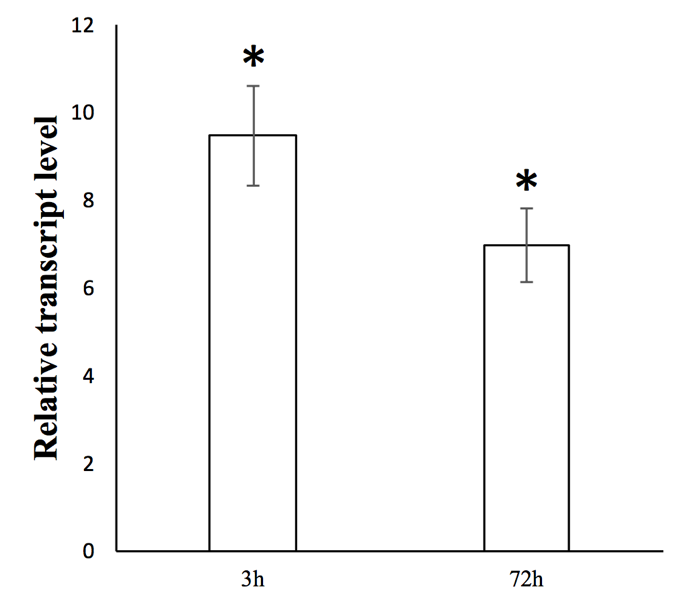
**

**Fig. S10.** Quantitative RT-PCR analysis of *OsPPCK2* after exposure of 25 μM cadmium for 3, and 72 hours. Statistical analyses of significant difference between control and cadmium- treated samples was carried out by Student’s T-test. Asterisks indicate significance (p < 0.05).

**Fig. S11**. The distributions of *Ka/Ks* ratio for functional categories within GSR genes compared to the genome median in rice and *Brachypodium distachyum*. (A) Bar diagram depicts the mean *Ka/Ks* ratio of the genome median and GSR genes. The error bar represents standard error (Unpaired t-test). (B) Boxplot represents the *Ka/Ks* ratio of different functional categories in GSR genes. The *Ka/Ks* mean value of GSR genes is represented by dotted line. The x-axis shows the functional categories and gene numbers in each category. Significance was calculated by comparing each category to total GSR genes. The error bar represents standard error (Unpaired t-test ; *, *p* < 0.1; **, *p* < 0.05; ***, *p* < 0.01).
